# Quantum algorithm for protein-ligand docking sites identification in the interaction space

**DOI:** 10.1007/s10822-025-00620-5

**Published:** 2025-07-05

**Authors:** Ioannis Liliopoulos, Georgios D. Varsamis, Theodora Karamanidou, Christos Papalitsas, Grigorios Koulouras, Vassilios Pantazopoulos, Thanos G. Stavropoulos, Ioannis G. Karafyllidis

**Affiliations:** 1 Department of Electrical and Computer Engineering, Democritus University of Thrace, Xanthi, 67100 Greece; 2 Pfizer Center for Digital Innovation, Thessaloniki, Greece; 3 Pfizer AIDA, Scientific Computing & HPC, Cambridge, USA

**Keywords:** Quantum computing, Quantum algorithms, Ligand docking, Drug design

## Abstract

Over the past two decades, the development of novel drugs evolved into a high-demanding computational field. There is a constant and increasing need for advanced methods for determining protein-ligand binding in the drug design process. Even after the introduction and use of High-Performance Computers in drug design, fundamental problems and constraints have not been dealt with in a satisfactory manner. This is partially due to the fact that ligand docking in proteins is a quantum mechanical process. With the quantum computers available today, the question “Can quantum computers be used in drug design and how?” arises naturally. A novel quantum algorithm for protein-ligand docking site identification is presented here. In detail, the protein lattice model has been expanded to include protein-ligand interactions. Quantum state labelling for the interaction sites is introduced, and an extended and modified Grover quantum search algorithm is implemented to search for docking sites. This algorithm has been tested and executed on both a quantum simulator and a real quantum computer. The results show that the quantum algorithm can identify effectively docking sites. The quantum algorithm is highly scalable and well-suited for identifying docking sites within large proteins, poised to harness the potential of increased quantum bits in the future.

## Introduction

Quantum computers utilize quantum mechanical properties, such as state superposition and entanglement to execute computations [1, 2]. Superposition and entanglement have no classical analogue and empower quantum computers to solve complex problems, which are impossible to solve using classical computers [3, 4]. The development of novel quantum algorithms for various applications constitutes a highly active research field [5, 6].

In Chemistry, quantum computing offers unprecedented opportunities for simulating molecular dynamics, elucidating electronic structures, and unraveling complex quantum phenomena [7]. Quantum algorithms, such as the Variational Quantum Eigensolver (VQE) [8] and the Quantum Phase Estimation (QPE) algorithm [9], offer efficient approaches to calculate molecular energies and properties with high accuracy and scalability. Significant progress has been made in developing quantum algorithms including algorithms for simulating chemical reactions [10], optimizing molecular geometries [11], and predicting molecular spectra [12].

Quantum computing in Biology and especially quantum algorithms in Bioinformatics offer new perspectives and potential [13]. Quantum computers can manipulate and analyze big datasets because of their inherent parallelism due to superposition. A quantum algorithm for de novo DNA sequence assembly, based on quantum walks has been developed and presented in [14]. Another quantum algorithm for reference guided DNA sequence alignment has been developed by quantizing the classical process of shift and compare, used in the classical approach [15]. A very interesting aspect of quantum computing in Biology is that biological processes can be accurately simulated using quantum computing techniques. It has been suggested that quantum computations could potentially be performed at the molecular level [16]. In another study the high efficiency of photosynthesis has been described using quantum transport [17]. Bacterial quorum sensing can be simulated using quantum gates [18].

Very promising advances in addressing the protein folding problem using quantum algorithms have also been made. A quantum annealing algorithm based on the quantum Ising model has been developed to encode and fold small chains on a lattice [19]. In addition, a quantum optimization approach based on the adiabatic model has been developed to find the lowest energy conformation of small amino acid chains in two dimensions [20]. Quantum walks, a universal model of quantum computation, have been applied to predict protein and peptide folding in three dimensions [21].

Drug design is a highly computationally intensive process. In the initial stages of drug discovery and design, a target protein of therapeutic significance is determined. Subsequently, Computer Aided Drug Design (CADD) algorithms are employed to locate potential docking sites within the protein’s structure. Additionally, these algorithms facilitate the search through extensive libraries of molecules to identify promising ligands via high-throughput screening [22]. Protein-ligand docking plays a pivotal role in drug discovery and design, allowing researchers to predict the binding mode and affinity of small molecules to target proteins. Classical computational approaches are molecular docking and molecular dynamics simulations using machine learning techniques [23, 24]. Identifying docking sites for drug discovery represents a computationally intensive task, necessitating solutions that can expedite this process [25].

The identification of ligand-binding sites on proteins is a fundamental task in structural biology, bioinformatics, and drug discovery. Algorithms for binding site prediction can be categorized into three main types: geometry-based, energy-based, and machine learning-based approaches. Geometry-based methods explore the protein structures to identify possible sites in which a ligand can bind. Approaches such as CASTp (Computed Atlas of Surface Topography of proteins) [26] and Fpocket [27] use spatial and surface properties to detect potential binding sites. Energy-based methods analyse molecular interactions to obtain the energetic favorability of potential binding sites. Algorithms such as Q-SiteFinder [28] use energy grids to predict binding sites based on interaction energies between the protein and ligands. Machine learning and deep learning approaches use large datasets of known protein-ligand complexes to train algorithms that accurately predict binding sites [29, 30].

Although discussions on the potential integration of quantum computers in drug design have been widely debated in the literature, the development of innovative quantum algorithms specifically for drug design has not been extensively explored [31, 32]. Here, we present a novel quantum algorithm for the protein-ligand docking site identification process in CADD. Since protein folding is essential in drug discovery, we use the protein lattice model, which was used for quantum protein folding in [19] and [20], as a starting point for our quantum algorithm. To include the ligand and protein interaction sites, we extend the protein lattice model by introducing a finer graining for the topology of interaction sites. Both protein and ligand interaction sites are represented by quantum registers comprising one quantum bit (qubit) for each type of interaction. Subsequently, a quantum search algorithm is employed to search for possible binding sites of the ligand to the protein. To achieve this, we modify and extend the well-known Grover quantum search algorithm [33]. We utilize quantum superposition by segmenting the protein into parts that are comparable to the ligand and set the protein interaction sites in superposition. The quantum algorithm is scalable, and larger proteins and ligands can be readily introduced as the number of available qubits increases. We ensure the quantum algorithm operates correctly by executing it on the Qiskit quantum simulator and deploying it on an actual quantum computer for validation. Our results demonstrate that the quantum algorithm can effectively identify docking sites and, further suggest that the adoption of quantum computing within CADD will significantly expedite the drug design process.

## The ligand-protein interaction space

The protein lattice model is an abstract model which has been extensively used in investigating protein properties and predict protein folding [34, 35]. In this model a two- or three-dimensional lattice is constructed with each protein amino acid occupying one vertex. Bonds between amino acids are represented as edges of the graph. The protein lattice model can be easily extended to three dimensions and can represent real proteins by increasing the density of the lattice, which in turn increases the representation resolution [36]. The protein lattice model has been utilized in quantum computational approaches to address the protein folding problem [19, 20]. In our approach, we do not consider the solvent effect on noncovalent interactions between proteins and ligand molecules. A quantum approach for simulating noncovalent interactions, including hydrophobic effects, has been developed and lays the foundation for more accurate modelling of such interactions in protein–ligand complexes [37].

**Fig. 1:**
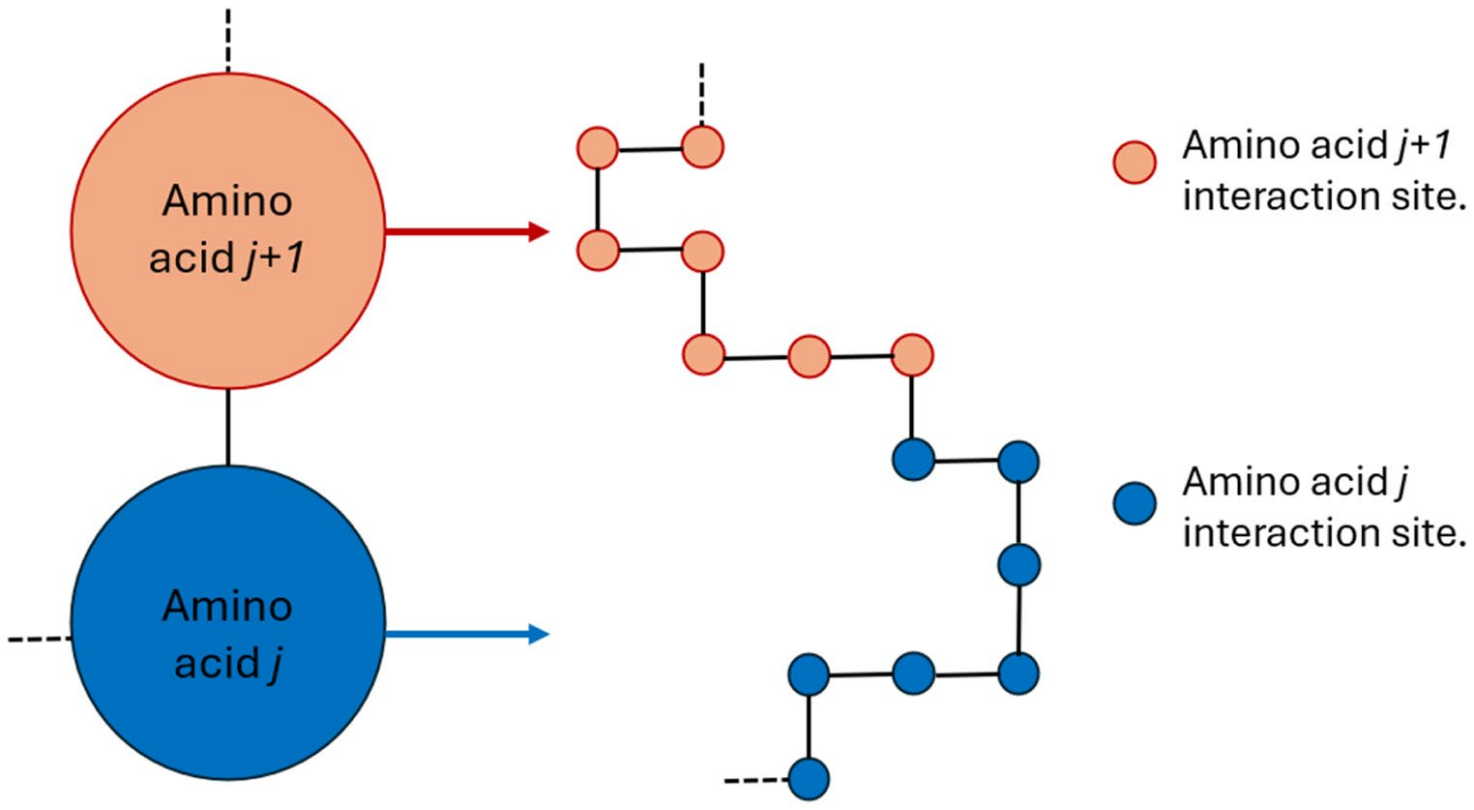
Schematic representation of the interaction space. Each amino acid is represented as an inner graph of its interaction sites. Amino acids are depicted by large circles and interaction sites with smaller ones

To introduce protein-ligand interactions, we extend the protein lattice model to include protein interaction sites. As depicted in Fig. 1, in the interaction space representation, each amino acid comprises a set of interaction sites, thus forming an inner lattice in which each interaction site occupies a vertex of the inner lattice, and the bonds between interaction sites are represented by the edges. On the left side of Fig. 1, two amino acids, *j* and *j + 1*, of the protein chain are shown (big circles). On the right side, the inner lattice of each amino acid is shown with the interaction sites having the same color as the corresponding amino acid (small circles). The interaction space is the space on which the quantum algorithm will act and evolve.

Turn-based encoding is an efficient approach to represent the locations of the interaction sites. This method employs a series of N-1 turns corresponding to the number of bonds [19, 20]. In this encoding, information about the position of interaction site is stored relative to the previous interaction site. Each turn taken by the j + 1 interaction site in the chain is encoded, requiring two qubits to uniquely represent one of the four directions (up, down, left, and right).

A systematic analysis of the frequency of common interactions of proteins and ligands observed in the Protein Data Bank showed that the most frequently used interactions are Hydrophobic and Hydrogen bonding followed by π-stacking, weak Hydrogen bonding, salt bridge, amide stacking and cation-π [38]. In the most general case, each interaction site must be represented by a quantum register comprising as many qubits as the interactions to be considered for ligand-protein binding. In contemporary quantum computers, which possess a limited number of qubits, only the two most frequently utilized interactions—hydrophobic interactions, and hydrogen bonding—are considered. This approach reduces the requisite number of qubits for each interaction site to two. The quantum algorithm has been devised in a manner that allows for the straightforward inclusion of additional interactions as the number of qubits expands.

**Fig. 2:**
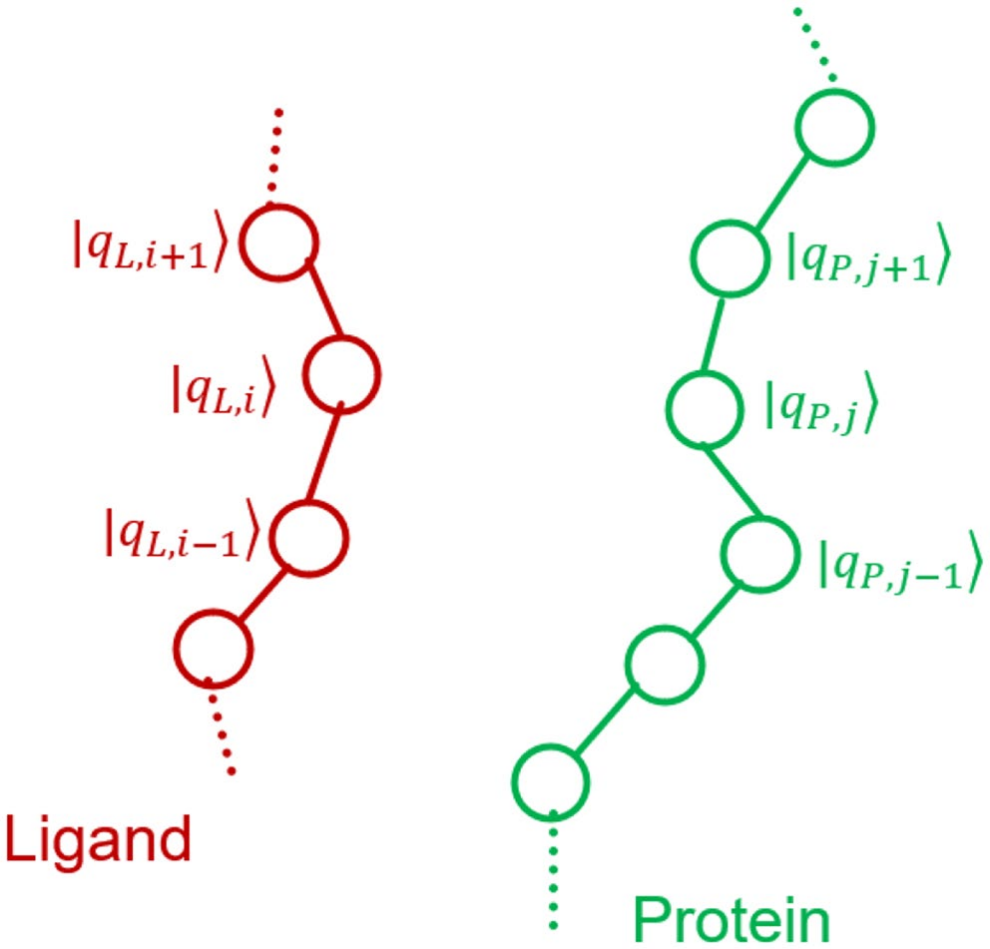
Interaction space model representation of ligands and proteins. Each lattice site state represents an interaction and constitutes the tensor product of two qubits: one for Hydrophobic interaction and one for Hydrogen Bond interactions

Figure 2 shows the interaction space representation of a ligand and a protein. By considering the two most frequently used interactions, namely the Hydrophobic and Hydrogen bonding, each interaction space site represents an interaction. Its state is the tensor product of two qubits, one for each interaction. The protein quantum state at site *j* is:

1 $$\:\begin{array}{c}|{q}_{P,j}\rangle =\:|{q}_{PH,j}\rangle \:\otimes\:\:|{q}_{PB,j}\rangle =\:|{q}_{PH,j}\:,\:\:\:{q}_{PB,j}\rangle \:\end{array}$$

In Eq. (1), $\:|{q}_{PH,j}\rangle \:$is the qubit describing the Hydrophobic interaction and $\:|{q}_{PB,j}\rangle$ the qubit describing the Hydrogen bonding interaction. The symbol H indicates the Hydrophobic interaction and B the Hydrogen bonding. The whole protein quantum state is given by the tensor product of its interaction sites:

$$\:|{q}_{P}\rangle =\cdots\:\:|{q}_{P,\:\:j-1}\rangle \:\otimes\:|{q}_{P,\:\:j}\rangle \:\otimes\:|{q}_{P,\:\:j+1}\rangle \cdots\:\:\Rightarrow\:$$

2 $$\matrix{|{q_P}\rangle = \cdots|{q_{PH,j - 1}}\,{q_{PB,j - 1}}\rangle \otimes|{q_{PH,j}}\,{q_{PB,j}}\rangle \hfill \cr \otimes|{q_{PH,j + 1}}\,{q_{PB,j + 1}}\rangle \hfill \cr} $$

The ligand quantum state at its *i*^*th*^ site is:

3 $$\:\begin{array}{c}|{q}_{L,i}\rangle =\:|{q}_{LH,i}\rangle \:\otimes\:\:|{q}_{LB,i}\rangle =\:|{q}_{LH,i}\:,\:\:\:{q}_{LB,i}\rangle \end{array}$$

In Eq. (3), $\:|{q}_{LH,i}\rangle$ is the qubit describing the Hydrophobic interaction and $\:|{q}_{LB,i}\rangle$ the qubit describing the Hydrogen bonding interaction. The whole ligand quantum state is given by the tensor product of its interaction sites:

$$\:|{q}_{L}\rangle =\cdots\:\:|{q}_{L,\:\:i-1}\rangle \:\otimes\:|{q}_{L,i}\rangle \:\otimes\:|{q}_{L,i+1}\rangle \cdots\:\:\Rightarrow\:$$

4 $$\matrix{|{q_L}\rangle = \cdots|{q_{LH,i - 1}}\,{q_{LB,i - 1}}\rangle \otimes|{q_{LH,i}}\,{q_{LB,i}}\rangle \hfill \cr \otimes|{q_{LH,i + 1}}\,{q_{LB,i + 1}}\rangle \hfill \cr}$$

The protein and ligand quantum states are the inputs to the quantum algorithm for searching possible docking sites.

## Quantum search

**Fig. 3:**
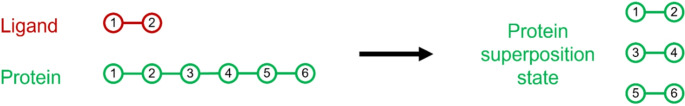
Graphical representation of the protein superposition state

To utilize the quantum advantage offered by the quantum superposition, we transform the protein state to a protein superposition state according to the ligand size as presented in Fig. 1. Each circle corresponds to an interaction site described by two qubits (Fig. 3).

Due to the nature of the problem, some of the protein interaction sites with similar properties, may occur multiple times. While we are searching whether an element is present, a unique occurrence satisfies this question and thus, we only consider the latest occurrence of each protein interaction site for the protein superposition state|s⟩.

For example:

$$\:|{q}_{L}\rangle =|1111\rangle $$

$$\eqalign{|{q_P}\rangle & =|1111110110001111\rangle \to|s\rangle \cr & = {1 \over {\sqrt 3 }}|1101\rangle + {1 \over {\sqrt 3 }}|1000\rangle + {1 \over {\sqrt 3 }}|1111\rangle \cr} $$

The probability amplitude of the states corresponds to the number of segmented parts of the protein been considered.

One of the most widely known quantum algorithms is Grover’s algorithm [39]. Its main advantage is that it offers a fast and effective approach for searching an element in an unsorted database. More specifically, let us suppose that we have a database consisting of *N* elements in completely random order and we want to retrieve a specific element from within this database. The most efficient classical algorithm would have to compare all the database’s elements one by one. Thus, its complexity is equal to $\:O\left(N\right)$. On the other hand, Grover’s algorithm, by utilizing quantum mechanical systems’ properties, such as the states’ superposition and the phase adjustment, would require $\:O\left(\sqrt{N}\right)$ recursions to find the desired element in that database.

For the quantum search part of our algorithm, we considered a modified version of Grover’s quantum algorithm. While Grover’s algorithm requires a uniform superposition of all basis states of the quantum register [40], the nature of the problem we are dealing with does not always satisfies this requirement. By utilizing the protein superposition state described above, the initial superposition state is:

5 $$\:\begin{array}{c}|s\rangle =\frac{1}{\sqrt{N}}{\sum\:}_{x=0}^{N-1}|x\rangle \end{array}$$

Where N is the number of unique occurrences of interaction sites in the protein state. This allows us to search in a smaller Hilbert space, i.e., a Hilbert subspace.

Our quantum search algorithm, just like Grover’s algorithm, consists of three main components· the target element $\:|{x}_{i}\rangle$, and the Oracle $\:\widehat{O}$ and Grover’s diffusion $\:\widehat{G}$ operators. The target element in our quantum search algorithm is the ligand state. All the aforementioned components are defined as follows:

The target element:

6 $$\:\begin{array}{c}|{x}_{i}\rangle =|ligand\rangle \end{array}$$

The Oracle operator:

7 $$\:\begin{array}{c}\widehat{O}=\:I\:-2\left|{x}_{i}\rangle \langle {x}_{i}\right|\end{array}$$

The diffusion operator:

8 $$\:\begin{array}{c}\widehat{G}=\:2\left|s\rangle \langle s\right|-I\end{array}$$

**Fig. 4:**
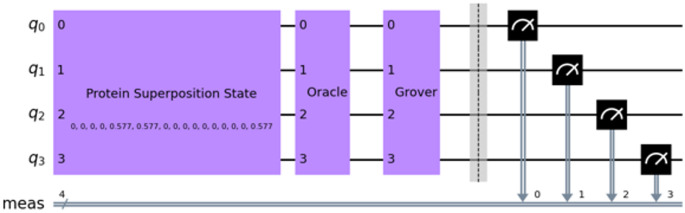
The quantum circuit of the search algorithm in Qiskit

To determine whether the target element is contained in the superposition state, fewer than $\:\sqrt{N}$ iterations are required because the search is performed in the Hilbert subspace containing only the basis states participating in the superposition. After the measurement of the quantum register, we get a probability distribution for all possible quantum states. To determine if the target element, i.e., the ligand, is present in the protein superposition state, we considered a threshold for the corresponding state’s probability. The threshold value is equal to 1/N, where N is the number of basis states in the superposition. This threshold derives from the extreme case where only two unique states are present in the protein superposition state. The more basis states in the superposition state, the greater the probability of the target element to be found after the measurement. The block form of the processes described above, developed in IBM’s Qiskit framework [41], is shown in Fig. 4.

## Quantum Euclidean distance

The Euclidean distance is a widely used metric and it is utilized mostly to examine the similarity degree between two objects that fall in the same class [42–44]. In mathematical terms it is expressed as the root of square difference between the co-ordinates of a pair of objects.

9 $$\:\begin{array}{c}{Euc{l}_{Dist}}_{XY}=\sqrt{\sum\:_{i=1}^{n}{\left({X}_{i}-{Y}_{i}\right)}^{2}}\end{array}$$

Within the scope of this work, we utilized the SWAP quantum test [45] to compute the Euclidean distance between two state vectors A and B [46]. These two vectors reflect the ligand and the potential protein docking site states, accordingly, as explained in detail in the next section.

First of all, we have to properly encode our data into quantum states. Here, we utilized the quantum amplitude embedding technique to properly encode our data. We will elaborate more on this in Sect. 5.1 of this work.

10 $$\:\begin{array}{c}|A\rangle =\:\frac{1}{\left|A\right|}\sum\:_{i}{A}_{i}|i\rangle \end{array}$$

11 $$\:\begin{array}{c}|B\rangle =\:\frac{1}{\left|B\right|}\sum\:_{i}{B}_{i}|i\rangle \end{array}$$

Then we define states $\:|\varPsi\:\rangle$ and $\:|\varphi\:\rangle$ as follows:

12 $$\:\begin{array}{c}|\varPsi\:\rangle =\:\frac{1}{\sqrt{2}}\left[|0\rangle \otimes\:\:|{\rm\:A}\rangle +\:|1\rangle \otimes\:\:|{\rm\:B}\rangle \right]\end{array}$$

13 $$\:\begin{array}{c}\:|\varphi\:\rangle =\:\frac{1}{\sqrt{{\rm\:Z}}}\left[\left|{\rm\:A}\right||0\rangle -\left|{\rm\:B}\right||1\rangle \right]\end{array}$$

Where $\:Z=\:{\left|A\right|}^{2}+{\left|B\right|}^{2}$. Afterwards, we calculate the distance between states $\:|\varPsi\:\rangle$ and $\:|\varphi\:\rangle$ using the SWAP test operator. To do so, we have to apply the appropriate quantum circuit and then measure the qubit 0 state. The corresponding circuit will be discussed further in Sect. 5.4. Finally, the Euclidean distance between vectors A and B is computed as follows:

14 $$\:\begin{array}{c}D=\:\sqrt{2Z{\left|\langle \varphi\:|\varPsi\:\rangle \right|}^{2}}\end{array}$$

### The quantum algorithm

**Fig. 5:**
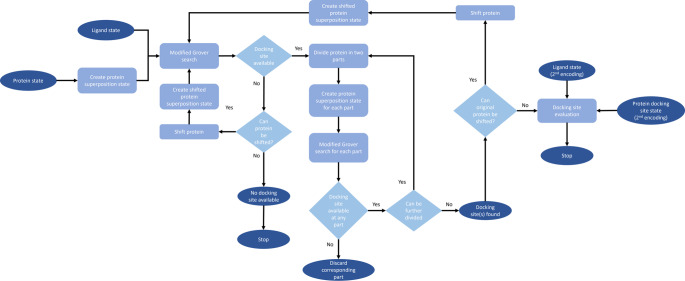
Quantum algorithm hierarchical flowchart. A high-resolution image can be found in the supplementary material

Our quantum algorithm consists of three main parts, namely the quantum search algorithm, the protein segmentation and shift and the identification and evaluation of docking sites part. The quantum search algorithm indicates whether there are any available potential docking sites, as well as their exact location, in side the given protein, as explained in detail in Sect. 3 of this work. The protein segmentation and shift part is used to examine all the possible docking sites within the protein. Finally, the evaluation of docking sites part, as the name implies, is used for the assessment of the potential docking sites regarding their similarity to the ligand. All the above can be summarized in the hierarchical flowchart of Fig. 5.

As mentioned in Sect. 2, we utilized two interactions of the ligand– protein interaction, i.e. “Hydrophobic interaction” and “Hydrogen bonding”, to describe each interaction site for both the ligand and the protein. These properties are described by continuous values which lie in a specific range bound by a minimum and a maximum value for each interaction. Thus, we considered two different encodings regarding the part of the problem they are referring to.

Hydrophobic interaction: $$\:[{h}_{min},{h}_{max}]$$

Hydrogen bonding: $$\:[{hb}_{min},{hb}_{max}]$$

### First encoding

The first encoding is related to the quantum search algorithm. To describe an interaction site we utilized two qubits, each one corresponding to each of the two interactions, namely Hydrophobic interaction and Hydrogen-bonds. Thus, a quantum state of an interaction site is given by:

15 $$\:\begin{array}{c}|q\rangle =|{q}_{h}\rangle \otimes\:|{q}_{hb}\rangle =|{{q}_{h}q}_{hb}\rangle \end{array}$$

where the left qubit corresponds to the hydrophobic interaction and the right one to the hydrogen bonding. The corresponding mapping of the properties’ continuous values to the quantum register is given by:

$$\:[{h}_{min},\frac{{{h}_{min}+h}_{max}}{2})\:\grave{a}\:\:|0\rangle $$

$$\:[\frac{{{h}_{min}+h}_{max}}{2},{h}_{max}]\:\grave{a}\:\:|1\rangle $$

$$\:{[hb}_{min},\frac{{{hb}_{min}+hb}_{max}}{2})\:\grave{a}\:\:|0\rangle \:$$

$$\:[\frac{{{hb}_{min}+hb}_{max}}{2},{hb}_{max}]\:\grave{a}\:\:|1\rangle \:$$

An example of a ligand and a protein quantum state is presented below:

Ligand state example:

$$\:|{q}_{L}\rangle =|{q}_{L,1}\rangle \otimes\:|{q}_{L,2}\rangle \otimes\:|{q}_{L,3}\rangle =|11\rangle \otimes\:|01\rangle \otimes\:|10\rangle =|110110\rangle $$

Protein state example:

$$\eqalign{|{q_P}\rangle &=|{q_{P,1}}\rangle \otimes|{q_{P,2}}\rangle \otimes|{q_{P,3}}\rangle \otimes|{q_{P,4}}\rangle \otimes|{q_{P,5}}\rangle \otimes|{q_{P,6}}\rangle \cr & =|11\rangle \otimes|11\rangle \otimes|10\rangle \otimes|01\rangle \otimes|10\rangle \otimes|10\rangle \cr & =|111110011010\rangle \cr} $$

### Second encoding

The second encoding is related to the evaluation of the potential docking sites, identified by the quantum search algorithm. In the same manner, we utilize one qubit for each corresponding interaction, except now we consider it to be in quantum superposition of its basis states.

$${\rm{Hydrophobic}}\,{\rm{interaction}}:[{h_{min}},{h_{max}}]\,\grave{a} \,a|0\rangle + b|1\rangle $$

$${\rm{Hydrogen}}\,{\rm{bonding}}:[h{b_{min}},h{b_{max}}]\,\grave{a} \,c|0\rangle + d|1\rangle $$

where a, b, c and d are the probability amplitudes and are given by:

16 $$\:\begin{array}{c}a=\:\frac{{h}_{max\:}-\:h}{\sqrt{{\left|{h}_{max}\:-\:h\right|}^{2}+{\left|{h\:-\:h}_{min}\right|}^{2}}}\end{array}$$

17 $$\:\begin{array}{c}b=\:\frac{{h-h}_{min\:}}{\sqrt{{\left|{h}_{max}\:-\:h\right|}^{2}+{\left|{h\:-\:h}_{min}\right|}^{2}}}\end{array}$$

18 $$\:\begin{array}{c}c=\:\frac{{hb}_{max\:}-\:hb}{\sqrt{{\left|{hb}_{max}\:-\:hb\right|}^{2}+{\left|{hb\:-\:hb}_{min}\right|}^{2}}}\end{array}$$

19 $$\:\begin{array}{c}d=\:\frac{{hb-hb}_{min\:}}{\sqrt{{\left|{hb}_{max}\:-\:hb\right|}^{2}+{\left|{hb-\:hb}_{min}\right|}^{2}}}\end{array}$$

Thus, a quantum state for one interaction site is:

$$\:|q\rangle \:=|{q}_{1}\rangle =\left(\:a|0\rangle +\:b|1\rangle \right)\otimes\:\left(\:c|0\rangle +\:d|1\rangle \right)=$$

20 $$\:\begin{array}{c}=\:\:ac|00\rangle +\:ad|01\rangle +\:bc|10\rangle +\:bd|11\rangle \end{array}$$

### Protein segmentation and shift

**Fig. 6:**
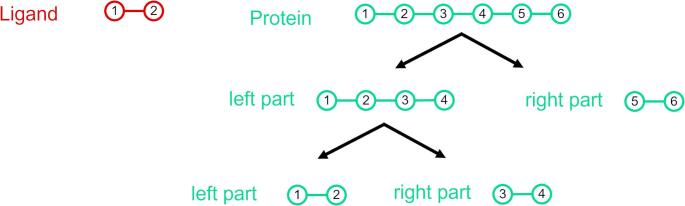
Protein segmentation according to the ligand size

To find the exact locations of the potential docking sites, we iteratively segment the protein described by the protein state into two parts as presented in Fig. 6. Again, we consider the size of the ligand to segment the protein.

For each segmented part of the protein, we utilize the protein superposition state that we have already described as an input to the modified Grover algorithm which we developed. The segmentation stops when the size of the ligand and the size, i.e. number of interaction sites, of the segmented protein parts are equal. At this point the algorithm deterministically indicates whether the corresponding interaction site is a potential docking site or not.

**Fig. 7:**
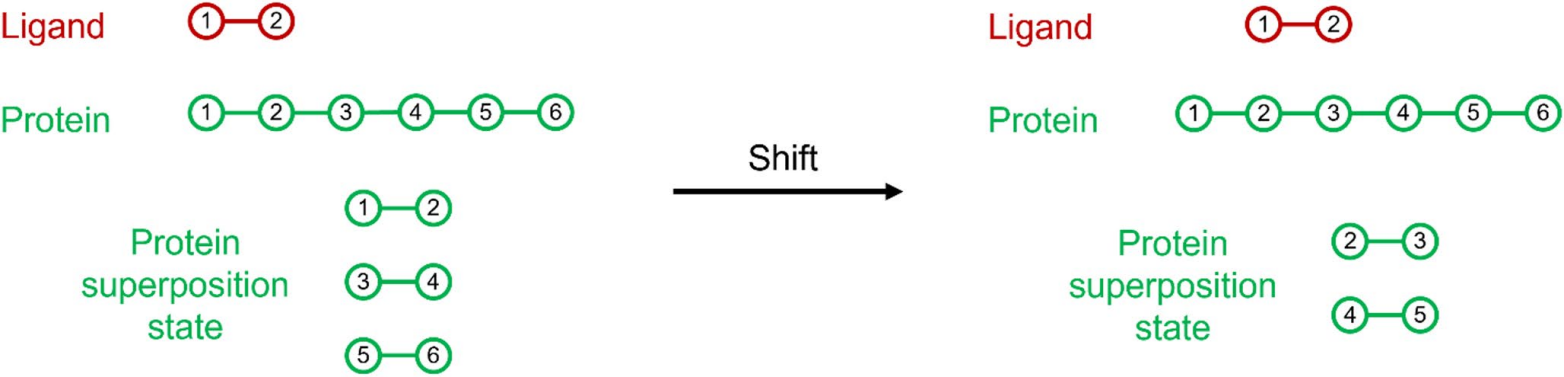
Protein shift example

During the creation of the quantum superposition state, we considered the size of the ligand and created the corresponding basis states, taking into account the first interaction site as the starting point. In order to cover all the combinations of neighboring interaction sites, we utilized a shift component to our algorithm that creates a ‘new’ shifted protein, as shown in Fig. 7.

For the newly created protein segmentation, the quantum search components will be applied, aiming to find additional potential docking sites. The shifting process is repeated iteratively until the ‘new’ created protein has not been a part of a previous search, or there are no more available interaction sites in the protein.

### Identification and evaluation of potential Docking sites

After the potential docking sites have been identified, we evaluate them regarding their similarity to the ligand. As mentioned before, each potential docking site is of the same size as the ligand. Therefore, the second encoding for the state labels is utilized to create quantum state vectors for the ligand and the potential docking sites. These vectors are the quantum superposition of the ligand and potential docking sites’ basis states and probability amplitudes according to the real continuous values of the Hydrophobic interaction and Hydrogen bonding properties.

A straightforward approach to compare the similarity of these vectors is the Euclidian distance, as mentioned in Sect. 4. The ligand superposition state acts as the benchmark vector while the potential docking sites superposition states are those compared with the ligand. The site which is closer to the ligand superposition state returns the smaller Euclidian distance and it corresponds to the best available docking site.

For this reason, we considered the quantized version of the Euclidian distance computation as mentioned in the relevant section.

In respect of the initialization of the quantum register, we consider the amplitude encoding and thus, the $\:|\varphi \:\rangle$ and $\:|\psi\:\rangle$ states are given by:

21 $$\:\begin{array}{c}|\varphi\:\rangle =\frac{1}{\sqrt{Z}}\left(\left|ligand\right|\left|0\rangle -\left|docking\_site\right|\right|1\rangle \right)\end{array}$$

Where:

22 $$\:\begin{array}{c}Z=\:{\left|ligand\right|}^{2}+{\left|docking\_site\right|}^{2}\end{array}$$

And

23 $$\:\begin{array}{c}|\psi\:\rangle =\:\frac{1}{\sqrt{2}}(|ligand\rangle \otimes\:|0\rangle \:+\:|docking\_site\rangle \otimes\:\left|1\rangle \right)\end{array}$$

By performing the swap test and measuring the control qubit, we acquire:

$$\:{P}_{cqubit}\left(0\right)=\:{\left|\frac{1}{2}\left[|\varPsi\:\varphi\:\rangle +|\varphi\:\varPsi\:\rangle \right]\right|}^{2}=\:\frac{1}{4}{\left|\left[|\varPsi\:\varphi\:\rangle +|\varphi\:\varPsi\:\rangle \right]\right|}^{2}=$$

24 $$\:\begin{array}{c}=\:\frac{1}{4}\left[\langle \varPsi\:\varphi\:|\varPsi\:\varphi\:\rangle +\langle \varPsi\:\varphi\:|\varphi\:\varPsi\:\rangle +\langle \varphi\:\varPsi\:|\varPsi\:\varphi\:\rangle +\langle \varphi\:\varPsi\:|\varphi\:\varPsi\:\rangle \right]\end{array}$$

The following relation has been used:

25 $$\:\begin{array}{c}\langle \varPsi\:\varphi\:|\varphi\:\varPsi\:\rangle =\:\langle \varPsi\:|\varphi\:\rangle \otimes\:\langle \varphi\:|\varPsi\:\rangle =\langle \varphi\:|\varPsi\:\rangle \otimes\:\langle \varPsi\:|\varphi\:\rangle \end{array}$$

Thus, based on Eq. 24, we derive the following:

26 $$\:\begin{array}{c}{P}_{cqubit}\left(0\right)=\frac{1}{2}+\frac{1}{2}\langle \varPsi\:|\varphi\:\rangle +\langle \varphi\:|\varPsi\:\rangle =\frac{1}{2}+\frac{1}{2}{\langle \varPsi\:|\varphi\:\rangle }^{2}\end{array}\:$$

By combining Eqs. 14 and 26, the Euclidian distance is given by:

27 $$\:\begin{array}{c}D=\:\sqrt{4Z\left({P}_{cqubit}\left(0\right)-0.5\right)}\end{array}\:$$

**Fig. 8:**
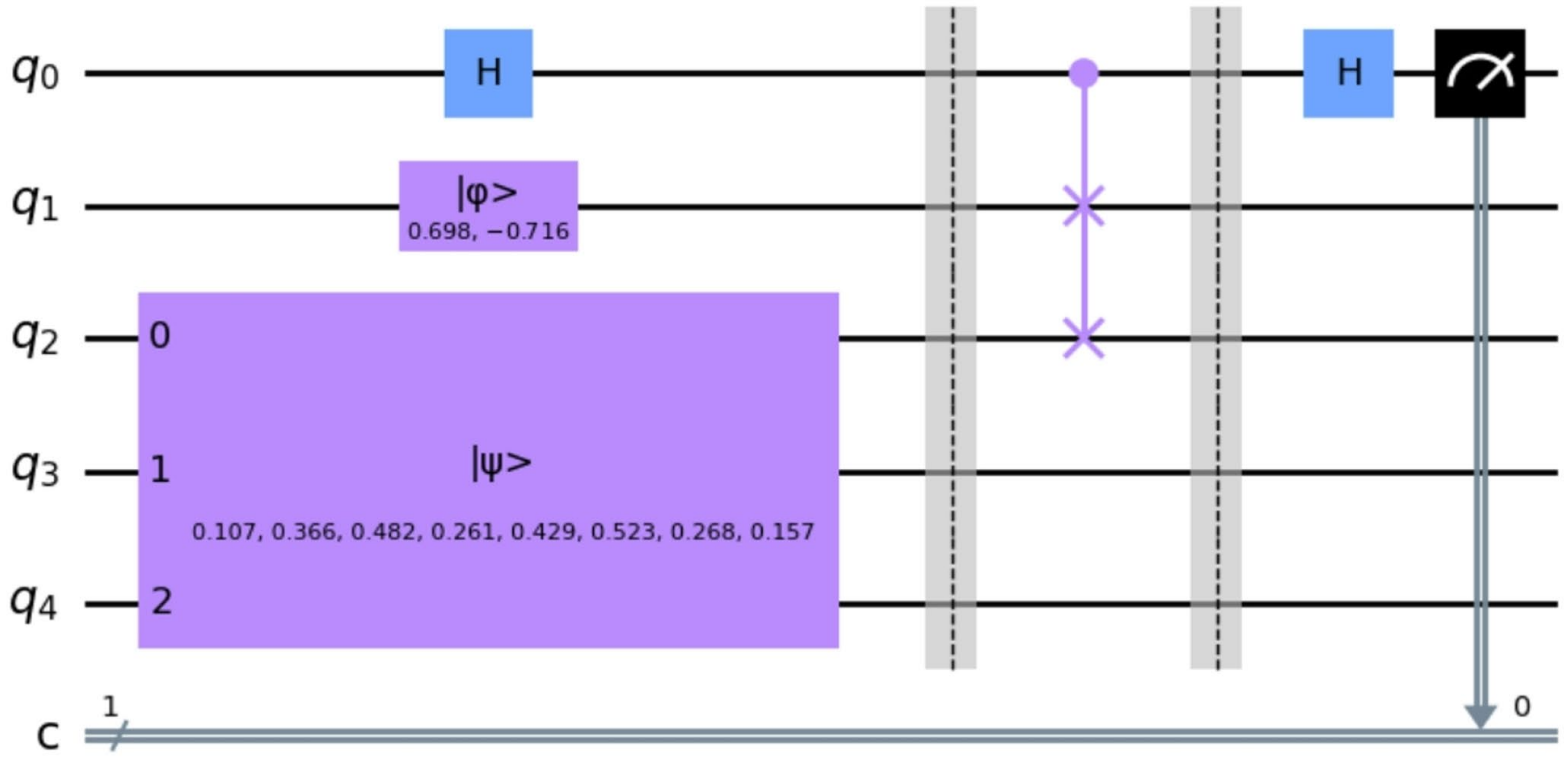
Qiskit quantum circuit for the evaluation of docking sites

A technical detail that is worth to be mentioned is that the distance computation is based on the probability of finding the control qubit in state $\:|0\rangle$. To improve the reliability of our results we opted for many shots on the quantum circuit execution and measurement. By doing this we reduced the statistical error percentage. The corresponding quantum circuit, as built in Qiskit, for the docking sites evaluation is shown in Fig. 8.

### Implementation of the quantum algorithm

Our quantum algorithm has been implemented in IBM’s Qiskit framework (https://www.ibm.com/quantum/qiskit). Qiskit is a reliable and easy-to-use environment to develop and test quantum algorithms. Moreover, not only it provides a plethora of simulators where anyone can benchmark his quantum algorithms/circuits but also provides a convenient way to execute any quantum algorithm/circuit to IBM’s quantum computers [41].

Regarding the quantum search algorithm, i.e. the modified Grover search, for the protein superposition state as well as the quantum oracle, we utilized Qiskit’s built-in StatePreperation() and UnitaryGate() methods, respectively. Considering the Noisy Intermediate-Scale Quantum era (NISQ), we designed the Grover diffusion operator using two approaches. Both approaches yield the same results on Qiskit’s simulators.

**Fig. 9:**
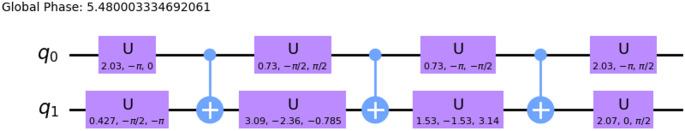
Unitary matrix transformed to quantum gates

In the first approach, we computed the Grover operator matrix, using Eq. 8, and utilized Qiskit’s built-in UnitaryGate() method to create the appropriate quantum gates, as shown in Fig. 9. The UnitaryGate() method transforms any unitary matrix to its corresponding quantum gates.

**Fig. 10:**
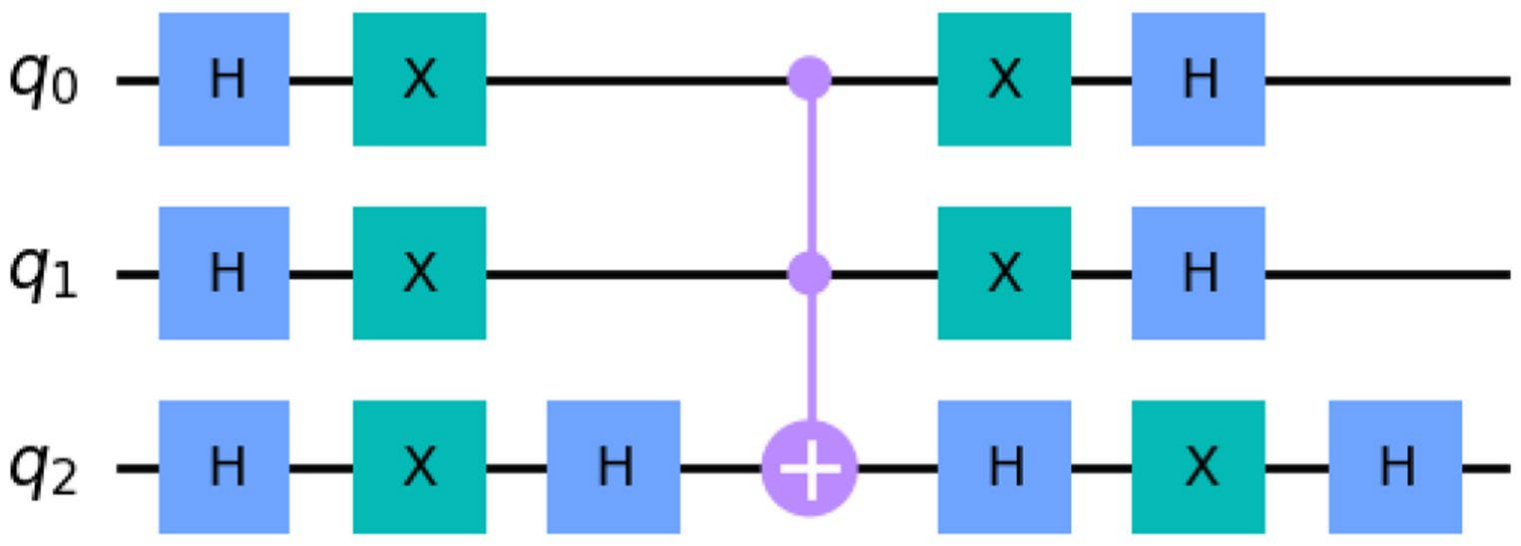
The Grover diffusion operator

**Fig. 11:**
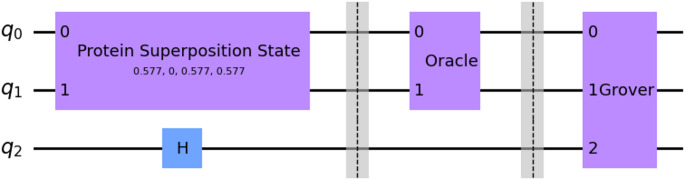
The Amplitude amplification quantum circuit

In the second approach, we opted for the amplitude amplification algorithm [47] to create the Grover diffusion operator as shown in Fig. 10. This approach requires an additional qubit for the quantum search circuit as presented in Fig. 11.

### Execution of the quantum algorithm on simulators

To test our algorithm’s capability to identify the location of the best docking site, we utilized the Sampler primitive that Qiskit provides. Moreover, we used appropriate values for the Hydrophobic interaction and Hydrogen bonding properties, for each one of the algorithm executions. All the following executions run on Qiskit’s simulators.

By utilizing the first quantum state encoding, the protein is shifted and segmented and the available docking sites are identified. While utilizing the second quantum state encoding, our algorithm identifies the best docking sites, as shown in Tables 1, 2 and 3.

**Table 1:**
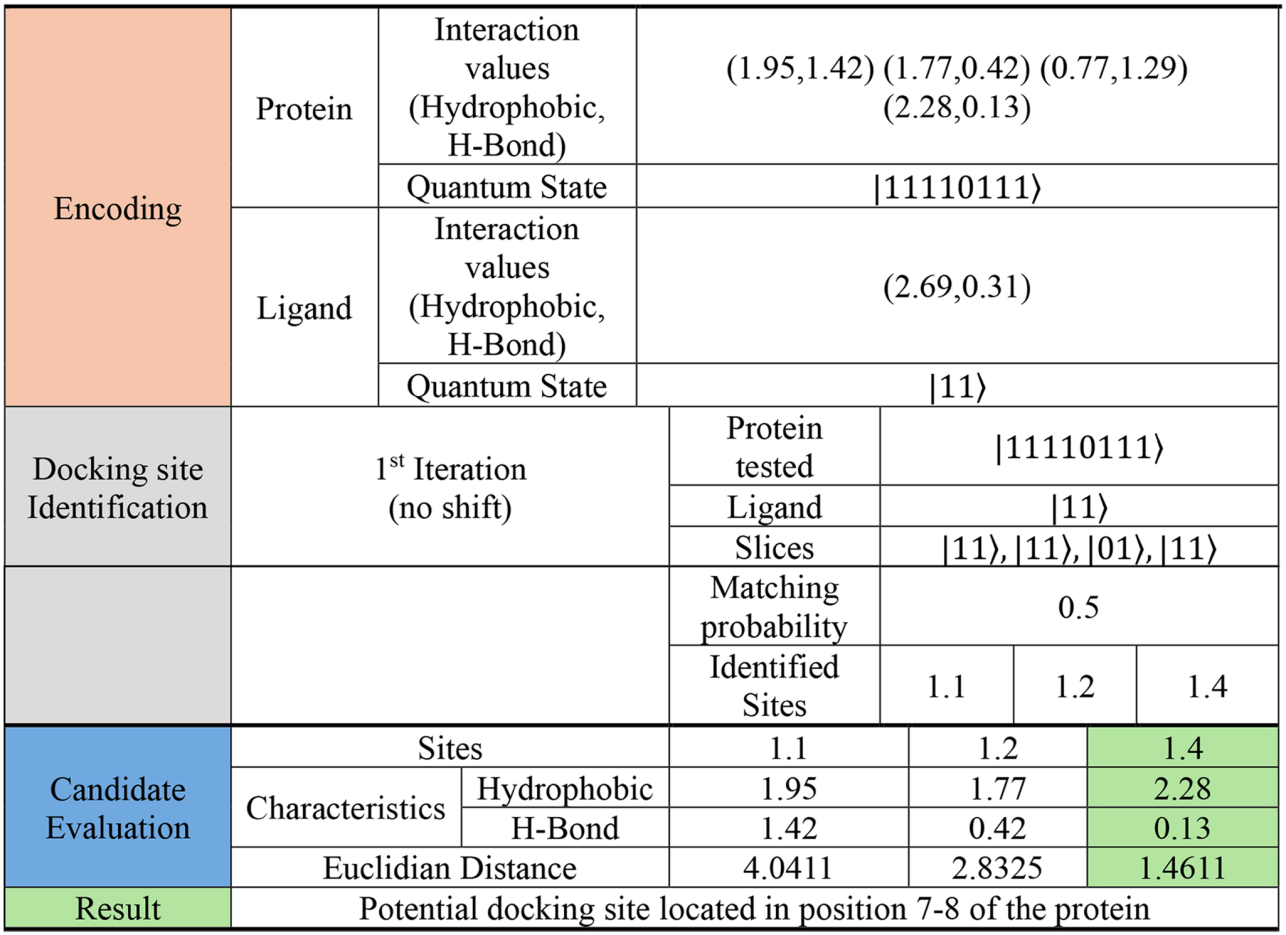
Algorithm execution results for a protein with 4 interaction sites and a ligand with 1 interaction site

**Table 2:**
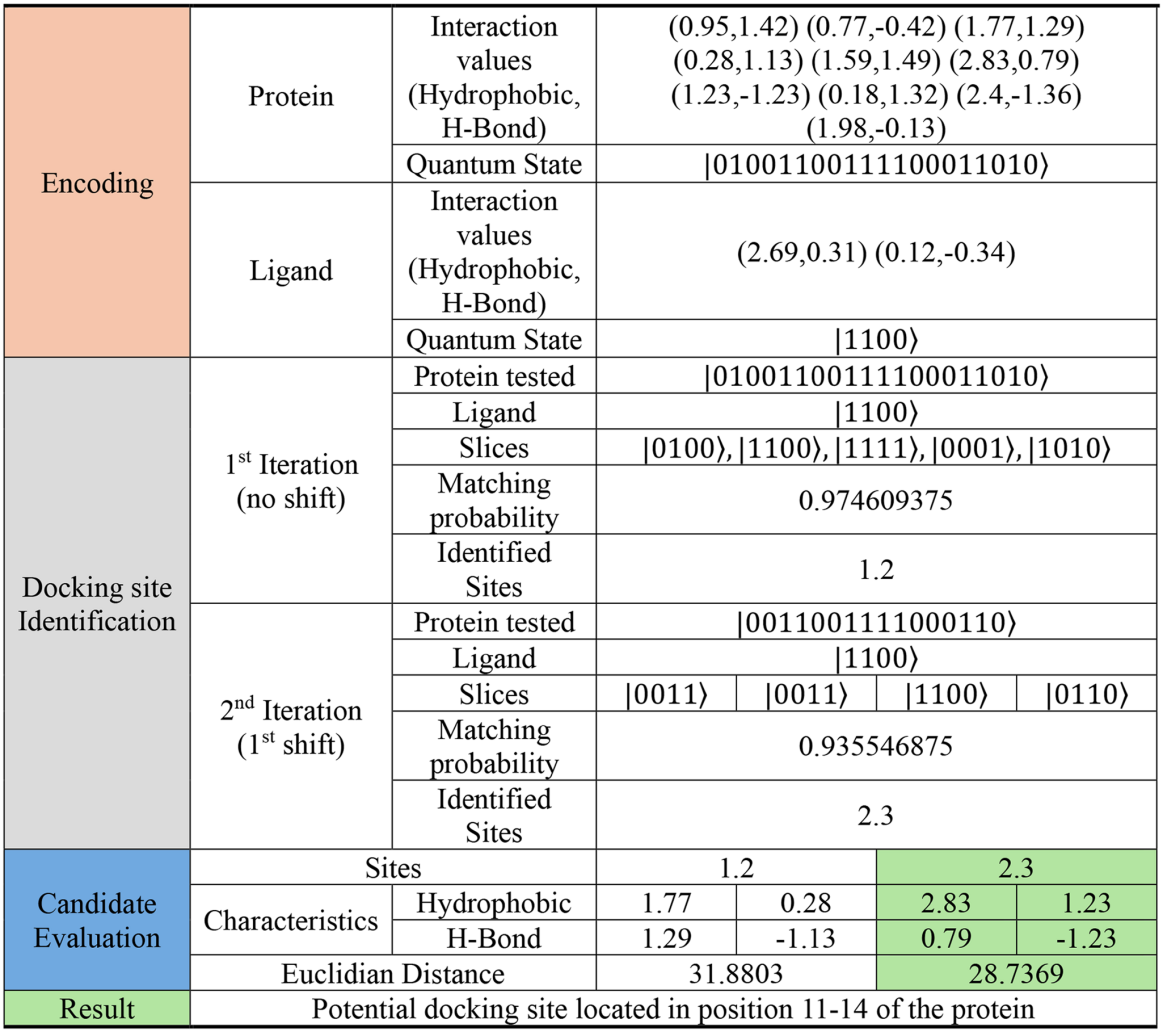
Algorithm execution results for a protein with 10 interaction sites and a ligand with 2 interaction sites

**Table 3:**
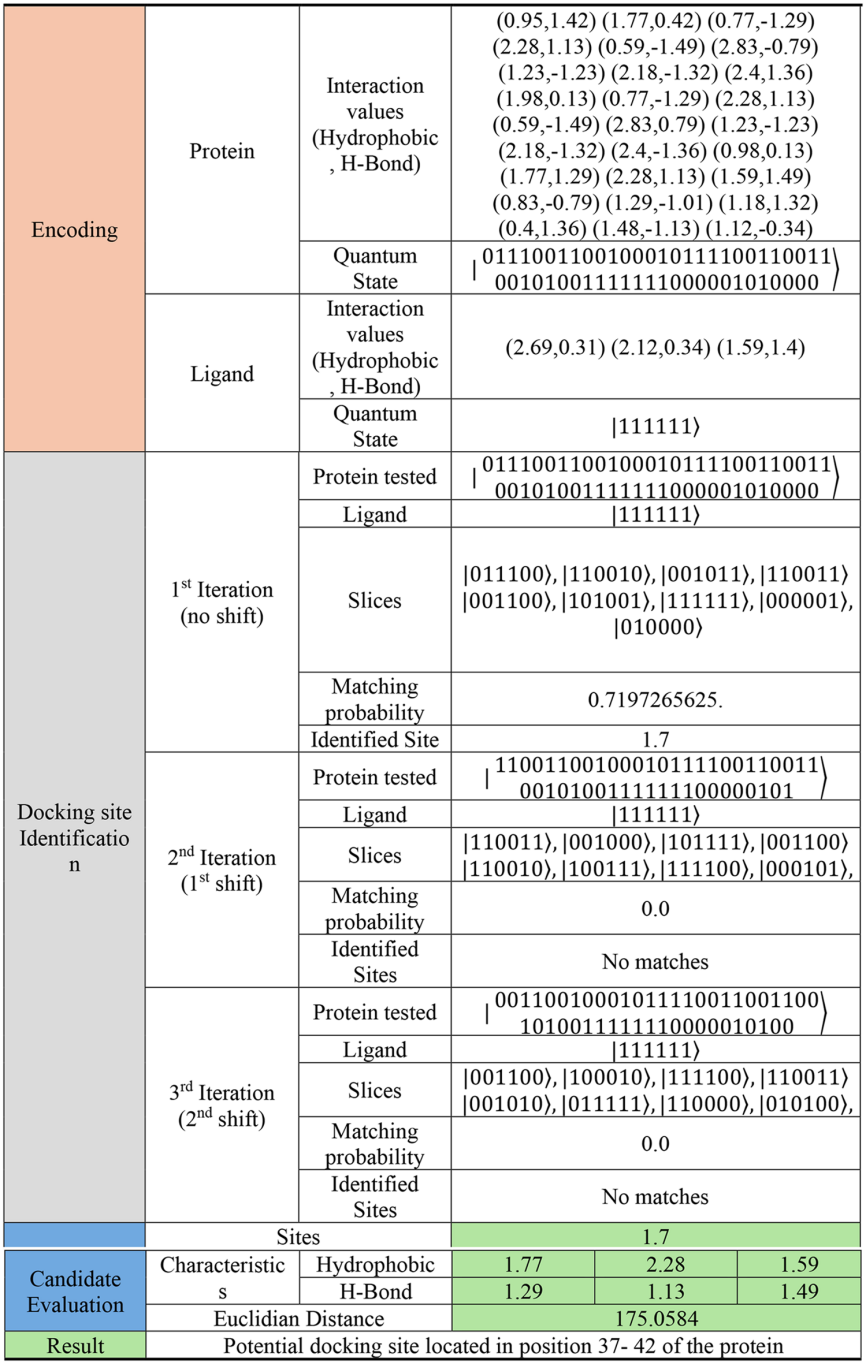
Algorithm execution results for a protein with 27 interaction sites and a ligand with 3 interaction sites

It is worth mentioning that our algorithm is scalable and designed in such a way that its scaling corresponds to the ligand size and with the presented examples the scalability of the quantum algorithm is demonstrated. Larger proteins can be searched for as the number of available qubits increases.

### Execution of the quantum algorithm on real IBM quantum computers

Apart from executing our quantum algorithm on Qiskit’s simulators, we also executed the algorithm on three real IBM quantum computers, all three comprising 127 qubits. More specifically, the quantum computers were the ‘ibm_brisbane’, ‘ibm_osaka’ and ‘ibm_kyoto’. We present the results of the first iteration, which involves searching the entire protein to identify the potential docking sites, for the ligand and protein given by:

$$\:|ligand\rangle =\:|1100\rangle $$

$$\:|protein\rangle =\:|010011001111000110100011\rangle $$

**Fig. 12:**
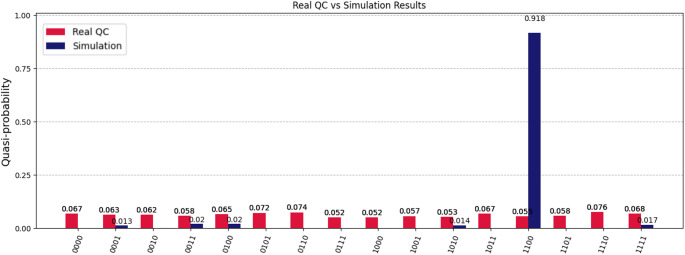
Comparative results of the quantum algorithm execution for a protein with 12 interaction sites and a ligand with 2 interaction sites on real IBM quantum computer (‘ibm_brisbane’) and on the Simulator. Quantum hardware errors affect the probability distribution

The expected probability of the state $\:|1100\rangle$ according to the Qiskit simulation was 0.918. Yet, due to noise in the real quantum computer the probability distribution was affected by errors and led to a small improvement as shown in Fig. 12.

**Fig. 13:**
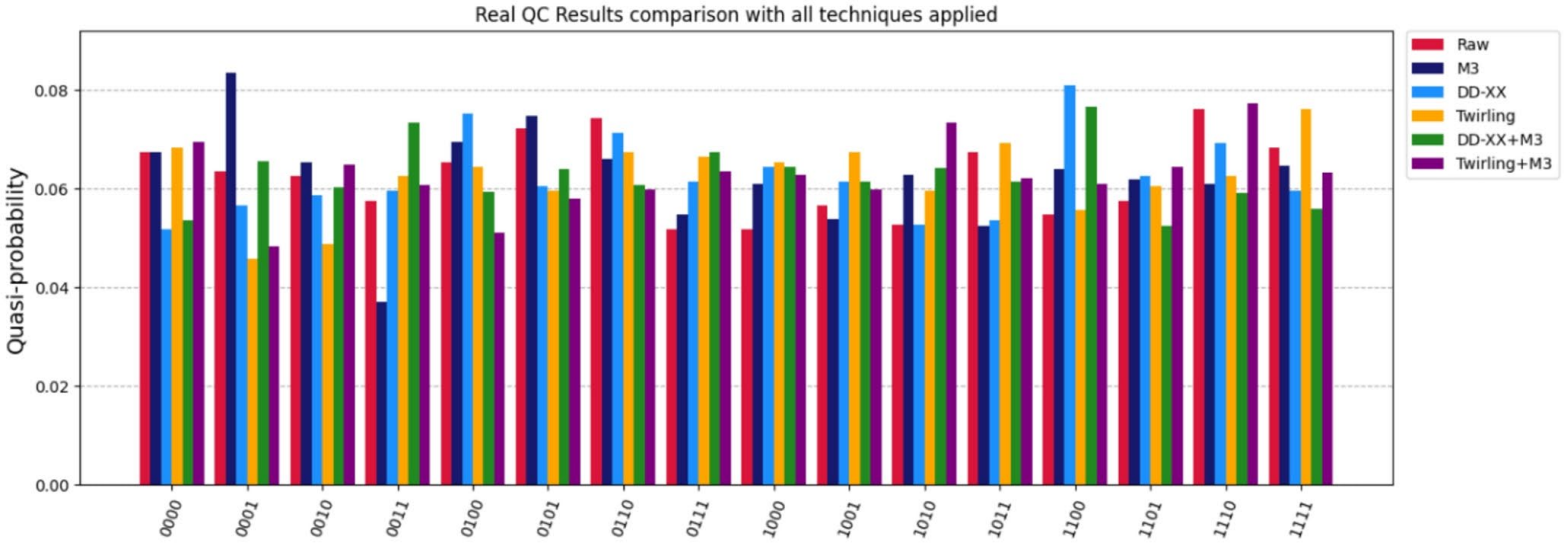
No-error (‘Raw’) and error mitigated results for the algorithm execution on ‘ibm_brisbane’. As depicted, either a single or a combination of the techniques mentioned were applied. The ‘DD-XX’ abbreviation stands for Dynamical Decoupling with XX pulse sequence. This technique yielded the best results overall, by eliminating a portion of the existing noise

We utilized two widely known quantum error mitigation techniques, namely the matrix-free measurement mitigation routine [48], known as M3, and the Pauli twirling technique [49], to improve the results. The former is a post-measurement technique for noisy input bitstring correction whereas the latter is a technique which reduces the coherent errors’ accumulation by converting arbitrary noise channels to Pauli channels during execution. Furthermore, to reduce coherent errors on idling qubits, during our quantum circuits’ execution, we applied the dynamical decoupling technique [50] with an XX pulse sequence. The hardware results show improvements, as shown in Fig. 13. Red color bars, denoted as ‘Raw’ in the legend, correspond to the original execution and all the others correspond to an error mitigated execution by applying a single or a combination of the techniques mentioned before. The best results were obtained by applying the dynamical decoupling technique during our algorithm execution.

Despite the hardware errors, the algorithm was executed correctly ensuring that the quantum algorithm will run without errors in the forthcoming fault-tolerant quantum computers.

## Conclusions

Within the scope of this research, a novel quantum algorithm was developed and introduced for the identification of protein-ligand docking sites. We expanded the existing protein lattice model to include protein-ligand interactions. In addition, we introduced a quantum state labelling for the interaction sites and implemented an extended and modified Grover quantum search algorithm to search for docking sites. Quantized Euclidean distance was used as a metric of the similarity degree between ligand and protein sites to identify the docking sites. The developed algorithm can identify a possible ligand docking site, multiple possible docking sites (if they exist), and can produce results indicating the absence of docking sites for a specific ligand. The quantum algorithm was successfully executed on IBM’s Qiskit quantum simulator and was executed on three real IBM quantum computers. Despite hardware decoherence errors, the algorithm produced results that ensure and verify its correctness. The quantum algorithm is shown to be scalable and can be used to identify docking sites in larger proteins as the available number of qubits increases, since quantum technology rapidly advances. Finally, the outcomes showcase that quantum computers have the potential to be used in the drug design process, significantly reducing the time required for the development and design of pharmaceuticals.

Currently available quantum computers comprise a limited number of qubits, which prevents the application of our method to real-world cases. To demonstrate our approach, we used the lattice protein model, which is commonly employed in the development of quantum algorithms for protein folding. Our primary focus was on formulating and encoding the problem in a manner suitable for quantum computation. The proposed quantum algorithm is scalable, and its extension to real-world cases will be straightforward once more qubits become available.

## Data Availability

No datasets were generated or analysed during the current study.

## References

[1] Yanofsky NS, Mannucci MA (2008) Quantum computing for computer scientists. Cambridge University Press, Cambridge. 10.1017/CBO9780511813887

[2] Hidary JD (2021) Quantum computing: an applied approach. Springer, Switzerland. 10.1007/978-3-030-23922-0

[3] Renner MJ, Brukner C (2022) Computational advantage from a quantum superposition of qubit gate orders. Phys Rev Lett 128:230503. 10.1103/PhysRevLett.128.230503 35749194 10.1103/PhysRevLett.128.230503

[4] Jozsa R, Linden N (2003) On the role of entanglement in quantum-computational speed-up. Proc. R. Soc. Lond. A.4592011–2032 10.1098/rspa.2002.1097

[5] Bharti K, Cervera-Lierta A, Kyaw TH, Haug T, Alperin-Lea S, Anand A, Degroote M, Heimonen H, Kottmann JS, Menke T, Mok WK, Sim S, Kwek LC, Aspuru-Guzik A (2022) Noisy intermediate-scale quantum algorithms. Rev Mod Phys 94. 10.1103/RevModPhys.94.015004

[6] Martyn JM, Rossi ZM, Tan AK, Chuang IL (2021) Grand unification of quantum algorithms. PRX Quantum 2:040203. 10.1103/PRXQuantum.2.040203

[7] McArdle S, Endo S, Aspuru-Guzik A, Benjamin SC, Yuan X (2020) Quantum computational chemistry. Rev Mod Phys 92(1):015003. 10.1103/RevModPhys.92.015003

[8] Peruzzo A, McClean J, Shadbolt P, Yung M-H, Zhou X-Q, Love PJ, O’Brien JL (2014) A variational eigenvalue solver on a quantum processor. Nat Commun 5:4213. 10.1038/ncomms5213 25055053 10.1038/ncomms5213 PMC4124861

[9] McClean JR, Babbush R, Love PJ, Aspuru-Guzik A (2017) The theory of variational hybrid quantum-classical algorithms. New J Phys 18(2):023023. 10.1088/1367-2630/18/2/023023

[10] Reiher M, Wiebe N (2017) Elucidating reaction mechanisms on quantum computers. Proc Natl Acad Sci USA 114(29):7555–7560 10.1073/pnas.1619152114 10.1073/pnas.1619152114 PMC5530650 28674011

[11] Ganzhorn M, Egger DJ, Barkoutsos P, Ollitrault P, Salis G, Moll N, Roth M, Fuhrer A, Mueller P, Woerner S, Tavernelli I, Filipp S (2019) Gate-efficient simulation of molecular eigenstates on a quantum computer. Phys Rev Appl 11(4):044092. 10.1103/PhysRevApplied.11.044092

[12] Babbush R, Aspuru-Guzik A (2018) Encoding electronic spectra in quantum circuits with linear T complexity. Phys Rev A 98(3):032309. https://link.aps.org/doi/10.1103/PhysRevX.8.041015

[13] Emani PS, Warrell J, Anticevic A et al (2021) Quantum computing at the frontiers of biological sciences. Nat Methods 18:701–709. 10.1038/s41592-020-01004-3 33398186 10.1038/s41592-020-01004-3 PMC8254820

[14] Varsamis GD, Karafyllidis IG, Gilkes KM, Arranz U, Martin-Cuevas R, Calleja G, Wong J, Jessen HC, Dimitrakis P, Kolovos P, Sandaltzopoulos R (2023) Quantum algorithm for de Novo DNA sequence assembly based on quantum walks on graphs. BioSystems 105037. 10.1016/j.biosystems.2023.105037 10.1016/j.biosystems.2023.105037 37734700

[15] Varsamis GD, Karafyllidis IG, Gilkes KM, Arranz U, Martin-Cuevas R, Calleja G, Dimitrakis P, Kolovos P, Sandaltzopoulos R, Wong J, Jessen HC (2023) Quantum gate algorithm for reference-guided DNA sequence alignment. Comput Biol Chem 107959. 10.1016/j.compbiolchem.2023.107959 10.1016/j.compbiolchem.2023.107959 37717360

[16] D’Acunto M (2023) Quantum computation by biological systems. IEEE Trans Mol Biol Multi-Scale Commun 9(2):257–262. 10.1109/TMBMC.2023.3272230

[17] Karafyllidis IG (2017) Quantum transport in the FMO photosynthetic light-harvesting complex. J Biol Phys 43:239–245. 10.1007/s10867-017-9449-4 28378262 10.1007/s10867-017-9449-4 PMC5471171

[18] Karafyllidis IG (2012) Quantum gate circuit model of signal integration in bacterial quorum sensing. IEEE/ACM Trans Comput Biol Bioinf 9(2):571–579. 10.1109/TCBB.2011.104 10.1109/TCBB.2011.104 21788673

[19] Perdomo-Ortiz A, Dickson N, Drew-Brook M et al (2012) Finding low-energy conformations of lattice protein models by quantum annealing. Nat Sci Rep 2:571. 10.1038/srep00571 10.1038/srep00571 PMC3417777 22891157

[20] Perdomo A, Truncik C, Tubert-Brohman I, Rose G, Aspuru-Guzik A (2008) Construction of model hamiltonians for adiabatic quantum computation and its application to finding low-energy conformations of lattice protein models. Phys Rev A 78:012320. 10.1103/PhysRevA.78.012320

[21] Varsamis GD, Karafyllidis IG (2023) A quantum walks assisted algorithm for peptide and protein folding prediction. BioSystems 104822. 10.1016/j.biosystems.2022.104822 10.1016/j.biosystems.2022.104822 36526010

[22] Gore M, Jagtap M (2023) U.B.: Computational Drug Discovery and Design. Springer Protocols. https://link.springer.com/book/10.1007/978-1-61779-465-0

[23] Durrant JD, McCammon JA (2011) Molecular dynamics simulations and drug discovery. BMC Biol 9(1):71. 10.1186/1741-7007-9-71 22035460 10.1186/1741-7007-9-71 PMC3203851

[24] Dhakal A, McKay C, Tanner JJ, Cheng J (2022) Artificial intelligence in the prediction of protein–ligand interactions: recent advances and future directions. Brief Bioinform 23(1). 10.1093/bib/bbab476 10.1093/bib/bbab476 PMC8690157 34849575

[25] Nantasenamat C, Prachayasittikul V (2015) Maximizing computational tools for successful drug discovery. Expert Opin Drug Discov 10(4):321–329. 10.1517/17460441.2015.1016497 25693813 10.1517/17460441.2015.1016497

[26] Dundas J, Ouyang Z, Tseng J, Binkowski A, Turpaz Y, Liang J (2006) CASTp: computed atlas of surface topography of proteins with structural and topographical mapping of functionally annotated residues. Nucleic Acids Res 34:W116–W118. 10.1093/nar/gkl282 16844972 10.1093/nar/gkl282 PMC1538779

[27] Le Guilloux V, Schmidtke P, Tufféry P, Fpocket (2009) An open source platform for ligand pocket detection. BMC Bioinformatics 10:168. 10.1186/1471-2105-10-168b 19486540 10.1186/1471-2105-10-168 PMC2700099

[28] Laurie ATR, Jackson RM (2005) Q-SiteFinder: an energy-based method for the prediction of protein–ligand binding sites. Bioinformatics 21(9):1908–1916. 10.1093/bioinformatics/bti315 15701681 10.1093/bioinformatics/bti315

[29] Jiménez J, Doerr S, Martínez-Rosell G, Rose AS, De Fabritiis G (2017) DeepSite: protein-binding site predictor using 3D-convolutional neural networks. Bioinformatics 33(19):3036–3042. 10.1093/bioinformatics/btx350 28575181 10.1093/bioinformatics/btx350

[30] Krivák R, Hoksza D (2018) P2Rank: machine learning-based tool for rapid and accurate prediction of ligand binding sites from protein structure. J Cheminform 10(1):39. 10.1186/s13321-018-0285-8 30109435 10.1186/s13321-018-0285-8 PMC6091426

[31] Cao Y, Romero J, Aspuru-Guzik CJ (2018) Potential of quantum computing for drug discovery. IBM J Res Dev 62(6). 10.1147/JRD.2018.2888987. pp. 6:1–6:20, 1

[32] Zinner M, Dahlhausen F, Boehme P, Ehlers J, Bieske L, Fehring L (2021) Quantum computing’s potential for drug discovery: Early-stage industry dynamics. Drug Discovery Today 26(7):1680–1688. 10.1016/j.drudis.2021.06.003 34119668 10.1016/j.drudis.2021.06.003

[33] Grover LK (1997) Quantum mechanics helps in searching for a needle in a haystack. Phys Rev Lett 79:325. 10.1103/PhysRevLett.79.325

[34] Hinds DA, Levitt M (1994) Exploring conformational space with a simple lattice model for protein structure. J Mol Biol 243(4):668. 10.1016/0022-2836(94)90040-X 7966290 10.1016/0022-2836(94)90040-x

[35] Kolinski A, Skolnick J (2004) Reduced models of proteins and their applications. Polymer 45:511–524. 10.1016/j.polymer.2003.10.064

[36] Bechini A (2013) On the characterization and software implementation of general protein lattice models. PLoS ONE 8(3):e59504. 10.1371/journal.pone.0059504 23555684 10.1371/journal.pone.0059504 PMC3612044

[37] Kaliakin D, Shajan A, Moreno JR, Li Z, Mitra A, Motta M, Johnson C, Saki AA, Das S, Sitdikov I, Mezzacapo A, Merz Jr KM (2024) Accurate quantum-centric simulations of supramolecular interactions, arXiv:2410.09209v2 10.48550/arXiv.2410.09209

[38] de Freitas FF, Scapira M (2017) A systematic analysis of atomic protein–ligand interactions in the PDB. Med Chem Commun. 8,1970 10.1039/C7MD00381A 10.1039/c7md00381a PMC5708362 29308120

[39] Grover LK (1996), July A fast quantum mechanical algorithm for database search. In Proceedings of the twenty-eighth annual ACM symposium on Theory of computing, pp. 212–219. 10.48550/arXiv.quant-ph/9605043

[40] Jozsa R (1999) Searching in Grover’s algorithm. 10.48550/arXiv.quant-ph/9901021

[41] Qiskit contributors.: Qiskit: An Open-source Framework for Quantum Computing (2023) 10.5281/zenodo.2573505

[42] Elmore KL, Richman MB (2001) Euclidean distance as a similarity metric for principal component analysis. Mon Weather Rev 129(3):540–549.

[43] Singh A, Yadav A, Rana A (2013) K-means with three different distance metrics. Int J Comput Appl 67(10). 10.5120/11430-6785

[44] Wang L, Zhang Y, Feng J (2005) On the Euclidean distance of images. IEEE Trans Pattern Anal Mach Intell 27(8):1334–1339. 10.1109/TPAMI.2005.165 16119271 10.1109/TPAMI.2005.165

[45] S. Pattanayak (2021) Quantum machine learning with Python using cirq from Google research and IBM Qiskit. Apress Chap 5, pp. 241–249

[46] Urgelles H, Picazo-Martínez P, Monserrat JF (2022), May Application of quantum computing to accurate positioning in 6 g indoor scenarios. In ICC 2022-IEEE International Conference on Communications, IEEE, pp. 643–647. 10.1109/ICC45855.2022.9838523

[47] Brassard G, Hoyer P, Mosca M, Tapp A (2002) Quantum amplitude amplification and Estimation. Contemp Math 305:53–74. 10.48550/arXiv.quant-ph/0005055

[48] Nation PD, Kang H, Sundaresan N, Gambetta JM (2021) Scalable mitigation of measurement errors on quantum computers. PRX Quantum 2(4):040326. 10.1103/PRXQuantum.2.040326

[49] Wallman JJ, Emerson J (2016) Noise tailoring for scalable quantum computation via randomized compiling. Phys Rev A 94(5):052325. 10.1103/PhysRevA.94.052325

[50] Ezzell N, Pokharel B, Tewala L, Quiroz G, Lidar DA (2023) Dynamical decoupling for superconducting qubits: A performance survey. Phys Rev Appl 20(6):064027. 10.1103/PhysRevApplied.20.064027

